# Newly Isolated *Kluyveromyces marxianus* Strain Capable of Producing Oleic Acid-Rich Oil for Human Consumption from Whey

**DOI:** 10.3390/foods15193556

**Published:** 2026-10-07

**Authors:** Alejandro Pérez-Rodríguez, Carlos Alberto Montenegro-Herrera, César Mateo Flores-Ortiz, Liliana Morales-Barrera, Eliseo Cristiani-Urbina

**Affiliations:** 1 Departamento de Ingeniería Bioquímica, Escuela Nacional de Ciencias Biológicas, Instituto Politécnico Nacional, Av. Wilfrido Massieu s/n, Unidad Profesional Adolfo López Mateos, Ciudad de México 07738, Mexico; alperezr@ipn.mx (A.P.-R.); cmontenegro@sinchi.org.co (C.A.M.-H.); lmoralesb@ipn.mx (L.M.-B.); 2 Departamento de Ciencias Básicas, Unidad Profesional Interdisciplinaria de Biotecnología, Instituto Politécnico Nacional, Av. Acueducto, Barrio La Laguna Ticomán, Ciudad de México 07340, Mexico; 3 Instituto Amazónico de Investigaciones Científicas Sinchi, Avenida Vásquez Cobo Entre Calles 15 y 16, Leticia 910001, Amazonas, Colombia; 4 Unidad de Biología, Tecnología y Prototipos, Facultad de Estudios Superiores-Iztacala, Universidad Nacional Autónoma de México, Avenida de los Barrios No. 1, Los Reyes Iztacala, Tlalnepantla de Baz 54090, Estado de México, Mexico; cmflores@unam.mx; 5 Laboratorio Nacional de Salud, Facultad de Estudios Superiores-Iztacala, Universidad Nacional Autónoma de México, Avenida de los Barrios No. 1, Los Reyes Iztacala, Tlalnepantla de Baz 54090, Estado de México, Mexico

**Keywords:** cheese whey, *K. marxianus*, microbial oil, monounsaturated fatty acids, oleic acid, oleaginous yeast, single-cell oil

## Abstract

Cheese whey is a widely available agri-food byproduct whose high organic load presents significant challenges for the dairy industry and environmental management. Its biovalorization into high-value, sustainable functional lipids is increasingly important given the environmental concerns associated with conventional vegetable oil production. This study examines the isolation, identification, and characterization of a wild-type yeast strain capable of synthesizing oleic acid-rich oil from cheese whey. Screening six different sources yielded thirteen microbial isolates capable of growing on whey lactose, of which five demonstrated neutral lipid accumulation. The isolate with the highest lipid content was selected and molecularly identified as *Kluyveromyces marxianus* CM3b. Batch cultivation of *K. marxianus* CM3b in whey-based medium reached a maximum biomass concentration of 5.50 ± 0.06 g/L and a lipid concentration of 2.68 ± 0.08 g/L at 24 h, corresponding to a lipid yield of 0.49 ± 0.02 g/g of biomass (approximately 49% *w*/*w*) and a 24-h volumetric lipid productivity of 2.68 ± 0.08 g/L·d. The extracted oil contained a high proportion of monounsaturated fatty acids (69.56%), predominantly oleic acid (53.51%), and a fatty acid profile comparable to those of avocado and olive oils. These findings highlight *K. marxianus* CM3b as a promising wild-type strain for the sustainable production of oleic acid-rich microbial oil from a whey-based substrate. However, further physicochemical characterization and strain-specific safety assessment are required to establish its suitability for food applications.

## 1. Introduction

The dairy industry is a cornerstone of the global agri-food sector. However, its ongoing expansion has resulted in the generation of substantial volumes of byproducts, which pose significant economic and environmental challenges. Among these byproducts, whey is particularly notable owing to the substantial volume generated during cheese and casein production, accounting for approximately 85–90% of the total volume of processed milk, with an estimated annual global output of approximately 162 million tons [1,2]. Despite containing a considerable fraction of the valuable nutrients of milk—including lactose, soluble proteins, vitamins, and minerals—a significant proportion of whey remains underutilized [3,4].

The main solute of whey is lactose, which makes up 70% to 80% of its total solids. This high lactose content is the primary contributor to whey’s critical environmental parameters, driving high chemical oxygen demand (60,000–80,000 mg/L) and biochemical oxygen demand (30,000–50,000 mg/L) [5]. Consequently, the improper discharge of untreated whey into soils, water bodies, or sewage systems causes accelerated oxygen depletion, foul odor formation, eutrophication, and severe alterations in aquatic and terrestrial ecosystems. Additionally, this practice not only disrupts wastewater treatment processes but also substantially increases purification costs [2,5,6,7].

From a biotechnological perspective, while its chemical composition makes cheese whey highly polluting when discharged without treatment into aqueous and terrestrial environments—having 100 times more polluting strength than domestic wastewater [2,7,8]—it also represents an outstanding, nutrient-rich substrate for microbial valorization into high-value compounds and functional ingredients [4,5,6]. This approach focuses on integrated biorefinery concepts to achieve simultaneous whey bioremediation and biovalorization and contributes to the principles of the circular bioeconomy [2,9].

Concurrently, there is an increasing worldwide demand for sustainable lipid sources as their applications are rapidly expanding in the food, nutraceutical, bioenergy, oleochemical, pharmaceutical, and cosmetic industries [4,10,11,12].

From a human nutrition and health perspective, lipids are essential macronutrients, serving as structural components of cell membranes, concentrated energy reserves, precursors of bioactive molecules (such as hormones), and carriers of fat-soluble vitamins. Notably, monounsaturated fatty acids (MUFAs), particularly oleic acid (C18:1), are essential components of human nutrition because of their well-known benefits [13,14,15]. Oleic acid, a non-essential omega-9 MUFA (C18:1 ω-9), is the predominant fatty acid (FA) in avocado and olive oils and a key component of the Mediterranean diet. It is widely associated with nutritional and physiological benefits, including favorable cardiovascular, immunological, neurological, and metabolic effects [13,14,15,16,17,18,19,20,21,22,23]. Consequently, identifying novel and sustainable sources of oleic acid-rich oils represents a significant challenge and opportunity for the food industry.

In this context, oleaginous microorganisms have emerged as promising alternatives to conventional lipid sources. When cultivated under optimal conditions, these microorganisms can accumulate at least 20% of their dry biomass as intracellular neutral lipids, predominantly in the form of triacylglycerols [1,24,25,26]. Notably, the FA profiles of microbial lipids—typically referred to as microbial or single-cell oils—closely resemble those of vegetable oils, facilitating their integration into existing food processing chains.

Microbial lipid production offers several advantages over conventional plant and animal sources, including rapid production cycles, high yields, reduced water and arable land requirements, independence from seasonal and climatic fluctuations, and the ability to utilize low-cost industrial byproducts or waste streams as substrates. Furthermore, microorganisms are amenable to genetic manipulation and metabolic engineering [4,27,28,29,30]. Lipid profiles can be further optimized by selecting appropriate microbial strains, refining culture conditions and production times, and precisely controlling fermentation parameters [29,31,32,33].

Among the various substrates investigated, cheese whey emerges as a particularly attractive option owing to its high abundance, widespread availability, and substantial lactose content. This makes it a rich carbon source that can be directly converted into lipids through specific metabolic pathways, either by employing microorganisms with innate lactose-metabolizing capabilities or by utilizing pretreatment strategies, such as enzymatic lactose hydrolysis [1,4]. Additionally, cheese whey contains soluble proteins, peptides, and micronutrients, which can reduce the need for external nutrient supplementation in the culture medium and enhance the overall economic feasibility of the process [6,7]. Consequently, the use of cheese whey not only facilitates bioremediation but also enables biovalorization, resulting in the production of high-value lipid ingredients with promising applications in the food and nutraceutical sectors.

Despite growing interest in microbial oils and whey valorization, further identification and characterization of wild-type oleaginous strains from microbial species with established food-related applications are needed to expand the production of health-promoting FAs from this substrate. In particular, the ability to utilize whey lactose does not necessarily imply an oil-producing phenotype, since lipid production and FA composition are highly dependent on the microbial strain and cultivation conditions [29,34]. Therefore, identifying new wild-type microbial strains with these characteristics may create additional opportunities to convert whey into value-added microbial oils. Likewise, comprehensive information integrating cell growth, lactose consumption, lipid production kinetics, and FA profiles of such food-grade microorganisms is limited. Therefore, the present study aimed to isolate and identify a wild-type oleaginous microorganism capable of converting a whey-based substrate into microbial oil. The study also sought to characterize the microorganism’s growth kinetics and lipid accumulation patterns and to evaluate the FA profile of the produced oil to assess its potential as a sustainable source of oleic acid-rich oil for food-related applications.

## 2. Materials and Methods

### 2.1. Culture Medium Preparation

Sweet whey powder (F & A Dairy Products, Inc., Las Cruces, New Mexico, USA) was utilized throughout this study to prepare the culture medium employed to enrich, isolate, purify, propagate, and culture microbial strains capable of growing on whey as a carbon and energy source. According to the supplier, the chemical composition of the sweet whey powder was as follows: ≥65% lactose, ≥11.5% crude protein, ≥0.5% crude fat, ≤0.25% crude fiber, and ≤8.5% ash.

The sweet whey powder was reconstituted with deionized distilled water to achieve a final lactose concentration of 58 g/L, which is within the range commonly reported for cheese whey (approximately 4–6%, *w*/*v*) [3,5,35,36].

To minimize protein interference during fermentation and subsequent oil extraction, the whey was deproteinized by thermocoagulation at 92 °C for 10 min, followed by filtration through filter paper. This process reduced the whey protein content from 10.46 ± 0.038 g/L to 0.766 ± 0.008 g/L [37], as quantified by the Bradford method [38] using bovine serum albumin as the calibration standard. Thermal deproteinization was selected because thermal treatments at temperatures around 90 °C are commonly used for whey protein recovery in the manufacture of whey cheeses such as Ricotta [39]. In addition, this treatment does not require chemical precipitating agents. Nevertheless, thermal deproteinization may also alter the composition of other whey constituents, potentially affecting microbial growth and lipid accumulation, which this work did not evaluate.

The deproteinized whey was supplemented with ammonium sulfate ((NH_4_)_2_SO_4_) and potassium dihydrogen phosphate (KH_2_PO_4_) at lactose/(NH_4_)_2_SO_4_ and lactose/KH_2_PO_4_ mass ratios of 4 and 20, respectively [35]. The resulting culture medium (referred to as whey medium hereinafter) was sterilized by autoclaving at 121 °C for 15 min. The initial pH of the whey medium was 6.5 ± 0.1.

### 2.2. Enrichment, Isolation, and Screening of Oleaginous Microorganisms

#### 2.2.1. Isolation Sources

To isolate microorganisms capable of growing on whey lactose and accumulating intracellular lipids, samples were collected from spoiled dairy products, spoiled dairy byproducts, and farm soils (Table 1).

#### 2.2.2. Batch Enrichment of Microbial Cultures

Batch enrichment cultures were established to selectively facilitate the growth of microorganisms capable of utilizing whey lactose as a sole carbon and energy source.

For the batch enrichment cultures, a small amount of each isolation-source sample was inoculated separately into 250 mL Erlenmeyer flasks containing 50 mL of sterile whey medium. The flasks were incubated at 150 rpm and room temperature (approximately 22 ± 2 °C) for 24 h under continuous agitation. To progressively enrich for lactose-assimilating microbial populations, 2% *v*/*v* of each microbial culture was used to successively inoculate Erlenmeyer flasks containing fresh culture medium, which were incubated under the aforementioned conditions, until the microbial cultures displayed stable macroscopic characteristics—such as color, turbidity, and opacity—indicative of microbial adaptation to the whey medium and enrichment of lactose-metabolizing populations. This batch enrichment process was performed for approximately 30 days.

#### 2.2.3. Isolation of Pure Microbial Cultures

The enriched microbial cultures were serially diluted tenfold to a final dilution of 10^−7^. Subsequently, 0.1 mL aliquots from the 10^−5^, 10^−6^, and 10^−7^ dilutions were aseptically spread onto whey agar plates and incubated at 30 ± 2 °C for 48 h. To obtain axenic cultures, individual microbial colonies were collected from the plates, and pure microbial isolates were obtained using the streak plate method. The stock cultures of the microbial isolates were maintained on whey agar plates at 4 °C and subcultured every two months to ensure their viability and stability.

#### 2.2.4. Screening of Oil-Accumulating Microbial Isolates by Oil Red O Staining

To preliminarily assess the lipid accumulation capacity of the isolates, each isolate was inoculated individually into Erlenmeyer flasks containing liquid whey medium and incubated at 150 rpm and 22 ± 2 °C for 48 h. Following incubation, microbial cells were collected by centrifugation and washed twice with deionized distilled water. The microbial cells were then stained with Oil Red O (ORO) (Merck KGaA, Darmstadt, Germany), which is a lipophilic azo dye with a high affinity for hydrophobic compounds that selectively stains neutral lipid inclusions, producing an intense red coloration, whereas other cellular components remain unstained or only faintly stained. The ORO staining method is a rapid, cost-effective technique widely employed in microbiological studies for the detection and visualization of intracellular neutral lipids. It is routinely used to qualitatively or semi-quantitatively screen the biotechnological potential of oleaginous microbial strains [40,41].

To capture the fluorescence color emitted by the ORO dye, lipid accumulation in microbial isolates was visualized using a ZEISS Axio Observer fluorescence microscope (Zeiss, Oberkochen, Germany) equipped with a rhodamine filter after staining the cells. *Yarrowia lipolytica*, a yeast known for its ability to accumulate lipids, was used as a positive control for lipid accumulation. In contrast, *Saccharomyces cerevisiae*, which does not accumulate lipids beyond the threshold considered oleaginous, served as a negative control. Both reference strains were subjected to the same staining and imaging conditions as the isolates.

Subsequently, positively tested microbial isolates were identified preliminarily through proteomic analysis.

#### 2.2.5. Preliminary Lipid Production Assessment of Microbial Isolates

To preliminarily and quantitatively evaluate the lipid-producing potential of the microbial isolates, each isolate (0.2 g/L) was individually inoculated into Erlenmeyer flasks containing liquid whey medium. The cultures were incubated at 150 rpm and 22 ± 2 °C with continuous shaking for 72 h. Samples were collected at 24-h intervals throughout the fermentation period to determine biomass and lipid production. The microbial isolate exhibiting the highest lipid accumulation was subsequently selected for further analysis and molecular identification.

### 2.3. Morphological Characterization and Microscopic Analysis of the Selected Microbial Isolate

The selected microbial isolate was cultivated on whey agar at 22 ± 2 °C for 48 h for macroscopic and microscopic characterization. Macroscopic colony morphology was evaluated based on colony shape, color, elevation, surface, margin, and consistency, whereas cellular morphology was examined by Gram staining and conventional optical microscopy.

For microscopic visualization of intracellular lipids, an isolated colony or a portion thereof was transferred onto a microscope slide containing 20 μL of 0.9% NaCl solution, homogenized, and spread over the slide. After air-drying at room temperature, the smear was heat-fixed, covered with 1 mL of ORO staining solution, and incubated in the dark for 15 min. The preparation was subsequently washed with phosphate-buffered saline (PBS) and examined by conventional optical microscopy using immersion oil. ORO-stained cells were also examined using a ZEISS Axio Observer fluorescence microscope equipped with a rhodamine filter.

To further visualize intracellular lipid accumulation in the selected microbial isolate, confocal laser scanning microscopy was performed. The selected isolate was cultivated for 72 h in 125-mL Erlenmeyer flasks containing 50 mL of whey medium. After incubation, the culture was centrifuged at 3500 rpm for 15 min, and the recovered biomass was washed with deionized distilled water to remove residual culture medium. An aliquot (100 μL) of biomass was mixed with 100 μL of ORO solution and allowed to stain for 5 min. Subsequently, 2 μL of the biomass–ORO suspension was placed on a microscope slide for confocal microscopy analysis. Images were acquired at the Center for Nanoscience and Micro- and Nanotechnologies of the National Polytechnic Institute (Centro de Nanociencias y Micro y Nanotecnologías del Instituto Politécnico Nacional) using a laser scanning confocal microscope (LSM 710 NLO, Carl Zeiss Meditec AG, Jena, Germany), at an excitation wavelength above 633 nm and 900× magnification. Images were acquired at a resolution of 1024 × 1024 pixels in RGB format.

### 2.4. Kinetic Study of Cell Growth, Lactose Consumption, and Oil Production by the Selected Microbial Isolate

To evaluate the kinetics of cell growth, lactose consumption, and lipid production by the selected microbial isolate, non-optimized batch fermentation assays were conducted in 1000 mL Erlenmeyer flasks containing 200 mL of whey-based culture medium. The flasks were inoculated with the isolate at an initial biomass concentration of 0.2 g/L. The flasks were agitated in an orbital shaker (Cole-Palmer, Vernon Hills, IL, USA) at 150 rpm and 22 ± 2 °C for 48 h. Samples were collected at 0, 4, 8, 12, 15, 20, 24, 30, 36, and 48 h of cultivation to determine biomass, lactose, and lipid concentrations. FA profiling of the microbial isolate oil was conducted at the incubation time corresponding to maximum lipid accumulation.

The performance of the microbial isolate in terms of growth, lipid, and FA production was assessed using the following criteria: (1) biomass yield based on lactose consumed (Y_XS,_ g of biomass/g of lactose), (2) volumetric biomass productivity (R_X_, g of biomass/L·d), (3) lipid yield based on biomass produced (Y_PX_, g of lipid/g of biomass), (4) volumetric lipid productivity (R_P_, g of lipid/L·d), (5) specific lipid productivity (R_PX_, g of lipid/g of biomass·d), (6) FA yield based on oil produced (Y_FAP_, mg of FA/mg of oil), (7) volumetric FA productivity (R_FA_, mg of FA/L·h), and (8) specific FA productivity (R_FAX_, mg of FA/g of biomass·h). These parameters were calculated as follows:

(1) $$Y_{XS}=\;\frac{X_{f}-\;X_{0}}{S_{0}-\;S_{f}}$$

(2) $$R_{X}=\frac{X_{f}-X_{0}\;}{t_{f}-t_{0}}$$

(3) $$Y_{PX}=\frac{P_{f}-P_{0}}{X_{f}-X_{0}}$$

(4) $$R_{P}=\frac{P_{f}-P_{0}}{t_{f}-t_{0}}$$

(5) $$R_{PX}=\frac{P_{f}-P_{0}}{{(t}_{f}-t_{0})(X_{f}-X_{0})}$$

(6) $$Y_{FAP}=\frac{{FA}_{f}-{FA}_{0}}{P_{f}-P_{0}}$$

(7) $$R_{FA}=\frac{{FA}_{f}-{FA}_{0}}{t_{f}-t_{0}}$$

(8) $$R_{FAX}=\frac{{FA}_{f}-{FA}_{0}}{\left(X_{f}-X_{0})(t_{f}-t_{0}\right)}$$

where $X_{0}$ and $X_{f}$ denote the biomass concentrations (g/L) at time $t_{0}=0$ d and $t=\;t_{f}$, $S_{0}$ and $S_{f}$ represent the lactose concentrations (g/L) at time $t_{0}=0$ d and $t=\;t_{f}$, $P_{0}$ and $P_{f}$ denote the oil concentrations (g/L) at time $t_{0}=0$ d and $t=\;t_{f}$, ${FA}_{0}$ and ${FA}_{f}$ represent the FA concentrations (mg/L) at time $t_{0}=0$ h and $t=\;t_{f}$, and $t_{f}$ denotes the fermentation time (h or d as indicated). These kinetic parameters provide a comprehensive assessment of microbial strain performance and serve as essential criteria for evaluating their suitability for potential oil production processes in industrial and food applications [26,29,42,43].

### 2.5. Analytical Techniques

#### 2.5.1. Determination of Biomass Concentration

Biomass concentration was determined as dry cell weight. Culture samples of known volume were filtered through pre-weighed Whatman GF/F glass microfiber filters (0.7 μm pore size; Cytiva, Amersham, UK). The filters containing retained biomass were washed twice with sterile distilled deionized water and subsequently dried at 60 °C until a constant weight was achieved. Biomass concentration (g/L) was calculated based on the volume of the filtered sample and weight differential of the glass microfiber filter before and after filtration and drying.

#### 2.5.2. Lactose Concentration Determination

Lactose concentration in culture samples was measured using an enzymatic assay involving β-galactosidase hydrolysis, followed by a glucose oxidase–peroxidase reaction, as described by Cristiani-Urbina et al. [35].

#### 2.5.3. Determination and Quantification of Total Oil Content

Total oil content was determined using the Folch method [44]. Following incubation, microbial cells were harvested by centrifugation at 3500 rpm for 15 min at room temperature, washed thrice with deionized distilled water, and then oven-dried at 60 °C to a constant cell dry weight. For extraction, 30 mg of dry biomass was mixed with 7.5 mL of chloroform–methanol solution (1:2 *v*/*v*) and sonicated for 1 h at 22 ± 2 °C. Next, 5 mL of chloroform and 1% NaCl solution (1:1 *v*/*v*) were added to the sonicated suspension to obtain a final chloroform–methanol–NaCl volume ratio of 2:2:1. The resulting mixture was centrifuged at 3000 rpm for 10 min to facilitate phase separation. The upper methanol–water layer was discarded, whereas the lipid-containing chloroform layer was collected and evaporated at 60 °C to a constant weight. Subsequently, the total oil content was determined gravimetrically and expressed as a percentage of dry biomass (% *w*/*w*).

#### 2.5.4. Fatty Acid Composition Analysis

FA composition of the extracted oils was analyzed using gas chromatography coupled with mass spectrometry following conversion of FAs to fatty acid methyl esters (FAMEs) using a 12% boron trifluoride–methanol solution, as described by Morrison and Smith [45]. FAMEs were separated and quantified using a gas chromatograph (Agilent 6850 Series II Network System) coupled to a mass spectrometer (Agilent Series 5975C) and equipped with a capillary column (Agilent 19091s-433E, 30 m × 250 μm × 0.25 μm) and a flame ionization detector (Agilent Technologies, Inc., Santa Clara, CA, USA). The oven temperature was initially held at 150 °C for 2 min, then increased to 200 °C at 5 °C/min, and then to 260 °C at 3 °C/min. The injector and detector temperatures were set at 220 and 290 °C, respectively. Helium served as the carrier gas at a constant flow rate of 1 mL/min.

#### 2.5.5. Proteomic Identification of the Microbial Isolates

The preliminary identification of the microbial isolates was performed by mass-spectrometry-based proteomics using a MALDI-TOF VITEK^®^ MS System (matrix-assisted laser desorption ionization time-of-flight mass spectrometry). Each isolate was analyzed using a VITEK^®^ MS apparatus (bioMérieux Vitek, Marcy l’Etoile, France) to obtain the mass spectral fingerprints of its ribosomal proteins. These spectra were compared against the VITEK MS In Vitro Diagnostic version 3.0.0 database and the Research Use Only version 14.12 database (Vitek MS Saramis database) to identify microbial isolates.

#### 2.5.6. Molecular Identification of the Selected Microbial Isolate

The molecular identification of the selected microbial isolate was conducted by the National Collection of Microbial Strains and Cell Cultures of the Center for Research and Advanced Studies of the National Polytechnic Institute (Colección Nacional de Cepas Microbianas y Cultivos Celulares del Centro de Investigación y de Estudios Avanzados del Instituto Politécnico Nacional). Identification was based on the amplification and sequencing of the 16S-ITS1-5.8S-ITS2-26S region using oligonucleotides ITS4 and ITS5, following the protocol described by White et al. [46].

### 2.6. Statistical Analysis

All experiments were conducted in at least duplicate. The kinetic data on the performance of the microbial isolates represent the average of at least four independent assays (n ≥ 4). Statistical analyses were performed using one-way ANOVA followed by Tukey’s multiple-comparison post hoc test in GraphPad Prism software, version 8.02 (GraphPad Software, Boston, MA, USA). Differences were considered statistically significant at *p* < 0.05. The results are presented as mean value ± standard deviation.

## 3. Results and Discussion

### 3.1. Isolation, Screening, Proteomic Identification, and Selection of Microbial Isolates Capable of Growing and Producing Lipids from Whey

Batch enrichment cultures were established to selectively promote the growth of microorganisms capable of utilizing whey lactose as a carbon and energy source. Following approximately 30 days of successive transfers in whey medium, stable microbial populations were established from each of the six isolation sources assayed.

Thirteen microbial isolates capable of growth on whey medium were obtained (Table S1), demonstrating their capacity to metabolize lactose under the conditions tested. However, only five of these microbial isolates—alongside the positive control (*Yarrowia lipolytica*)—demonstrated intense red intracellular inclusions when stained with ORO and examined under fluorescence microscopy, indicative of intracellular neutral lipid accumulation, whereas the negative control (*Saccharomyces cerevisiae*) showed no detectable ORO-associated fluorescence. These ORO-positive microbial isolates (CM1a, CM2a, CM3a, CM3b, and CM5a) were designated by combining the identification code of the isolation source (e.g., CM1 and CM2) with a distinguishing letter (a or b) for microbial isolates from the same isolation source (e.g., CM3a and CM3b, both derived from isolation source 3, spoiled unpasteurized whey; refer to Table 1 and Table S1). The ORO-positive isolates CM1a, CM2a, CM3a, CM3b, and CM5a were subsequently subjected to proteomic identification and quantitative evaluation of lipid production in whey medium.

These results suggest that lactose metabolism and oil accumulation are physiologically independent traits, since not all lactose-utilizing microorganisms are oleaginous. This observation aligns with the existing literature, which emphasizes that lipid accumulation is a strain-specific characteristic among microorganisms.

Overall, the targeted screening for intracellular oil-accumulating microorganisms was critical for identifying isolates with genuine potential for microbial oil production, rather than merely selecting lactose-utilizing isolates.

The microbial isolates CM1a, CM2a, CM3a, CM3b, and CM5a were identified as *Candida krusei*, *Candida krusei*, *Candida tropicalis*, *Candida kefyr*, and *Pichia fabianii*, respectively, by proteomic analysis, each demonstrating 99.99% similarity (Table 2).

To assess the lipid production potential of these yeast isolates, each was cultured individually in whey medium for 72 h. The total lipid content (lipid percentage of dry biomass weight or lipid yield) for the five microbial isolates at three fermentation times is presented in Table 3.

Among all isolates tested, *C. kefyr* CM3b showed the highest lipid accumulation, reaching 50.03 ± 2.96% (*w*/*w*) after 24 h of fermentation. This value was higher than those observed for *C. tropicalis* CM3a (41.77 ± 3.12%), *C. krusei* CM1a (33.14 ± 2.31%), *C. krusei* CM2a (32.67 ± 3.31%), and *P. fabianii* CM5a (16.25 ± 0.91%) at their respective peak production times (48 h).

According to established criteria, microorganisms that accumulate more than 20% lipids in their dry biomass are classified as oleaginous [26,43]. *C. kefyr* CM3b clearly meets this criterion, with a lipid content nearing 50%. However, it is important to note that the lipid accumulation dynamics observed in this case did not entirely align with the typical patterns seen in oleaginous yeasts. Usually, lipid storage in these yeasts continues throughout the stationary phase in response to nutrient stress and an excess of organic carbon [26,43].

Notably, *C. kefyr* CM3b reached its maximum lipid accumulation within 24 h of incubation, whereas the other yeast isolates required 48 h to achieve peak lipid levels. The relatively short cultivation time required to reach maximum lipid accumulation under the evaluated conditions is a potentially favorable characteristic of *C. kefyr* CM3b that may reduce processing time and improve volumetric lipid productivity. However, process optimization and controlled bioreactor studies are needed to determine whether these potential benefits hold under larger-scale cultivation conditions.

Furthermore, although *C. krusei* CM1a, *C. krusei* CM2a, and *C. tropicalis* CM3a demonstrated promising lipid-producing capabilities, these yeast species are recognized as opportunistic pathogens, particularly posing risks to immunocompromised or vulnerable individuals [47,48,49,50,51]. Consequently, the application of these yeast strains in food or nutraceutical oil production is constrained by regulatory and safety considerations.

By contrast, *C. kefyr*, the preliminary identification assigned to isolate CM3b by MALDI-TOF MS, has historically been recognized as the anamorphic (asexual) form of *Kluyveromyces marxianus* and is currently regarded as a synonym of this species [52,53]. Therefore, the preliminary identification of isolate CM3b as *C. kefyr* was taxonomically consistent with its subsequent molecular assignment to *K. marxianus*, as described in Section 3.2.

Unlike the other yeast species identified during screening, *K. marxianus* has an established history of use in food fermentations, making the isolate CM3b particularly interesting for further investigation of microbial oil production for potential food applications [54,55,56].

Based on the aforementioned results, isolate CM3b showed the highest lipid yield and the shortest lipid production time. Consequently, this yeast strain was selected for further molecular identification, morphological and microscopic characterization, and kinetic characterization.

### 3.2. Molecular Identification of Yeast Isolate CM3b

Molecular identification of yeast isolate CM3b was performed by amplifying the 16S-ITS1-5.8S-ITS2-26S rDNA region. The resulting polymerase chain reaction (PCR) product was sequenced and edited, generating a consensus sequence of 694 nucleotides (Figure 1).

The obtained sequence was compared with those deposited in GenBank using the BLAST tool (version 2.7.1) provided by the National Center for Biotechnology Information. The analysis revealed 100% sequence coverage and 100% identity with *Kluyveromyces marxianus* strain Rubing Y-13 (accession number: LC269188.1), with an E-value (expect value) of 0.0, supporting the identification of isolate CM3b as *K. marxianus*. This molecular assignment is consistent with the preliminary MALDI-TOF MS identification of CM3b as *C. kefyr*, which is recognized as the anamorphic name and a synonym of *K. marxianus* [52,53].

To further support this identification, a phylogenetic dendrogram was constructed using the neighbor-joining method (Tamura 3-parameter model) with 1000 bootstrap replicates. The analysis placed isolate CM3b within the *K. marxianus* clade (Figure 2), providing additional support for its taxonomic assignment.

The identification of isolate CM3b as *K. marxianus* is relevant to its potential food biotechnology applications. This yeast species has an established history of use in food fermentations (cheese, yogurt, and kefir) and is included in the European Food Safety Authority’s (EFSA) Qualified Presumption of Safety (QPS) framework. Furthermore, *K. marxianus* strains hold Generally Recognized as Safe (GRAS) status from the U.S. Food and Drug Administration (FDA) [54,57,58]. However, species-level recognition and GRAS determinations for particular uses do not establish the safety of every newly isolated strain or its derived products. Because this study did not include strain-specific safety tests, additional safety characterization of *K. marxianus* CM3b and its oil will be required before establishing their suitability for food applications. Nonetheless, using a wild-type strain from a yeast species with established food-related applications provides a solid, relevant basis for further investigation of microbial oil production.

From an industrial perspective, *K. marxianus* possesses several physiological traits that are highly advantageous for fermentation processes. (1) It can grow on a broad spectrum of substrates, including whey, other dairy by-products, lactose, inulin, and pentose sugars [54,59]. (2) It demonstrates significant thermotolerance, capable of growing and fermenting at temperatures up to 52–55 °C and 45 °C, respectively [60,61]. (3) It has a high growth rate and is the fastest-growing eukaryotic microorganism known [59]. (4) It is predominantly Crabtree-negative, which is particularly beneficial for lipid production, as it facilitates respiratory metabolism and efficient carbon flux distribution toward biomass and metabolite synthesis rather than ethanol formation [54,58,61]. (5) It falls within the QPS framework in Europe and has multiple GRAS-noticed applications in the United States, making it particularly suitable for commercial applications in the food, feed, and pharmaceutical industries [54]. (6) It possesses established probiotic health benefits and valuable properties for bakery applications [54,56].

Although *K. marxianus* has been extensively investigated for the production of single-cell protein, various enzymes (including inulinase, β-galactosidase, pectinases, and lipases), bioethanol, a diverse array of volatile organic compounds contributing to the aroma profiles of foods and beverages, γ-aminobutyric acid, lactic acid, fructo-oligosaccharides, and heterologous proteins [54,55,56,62], reports on lipid production by wild-type strains remain limited. Previous studies have documented lipid production by *K. marxianus* IG1 in peptone and yeast extract media supplemented with glucose, lactose, or glycerol, as well as in deproteinized potato wastewater with similar supplements [63]; by an engineered *K. marxianus* strain in defined media [59]; and by *K. marxianus* ATCC 64884 grown on electro-activated whey [64]. However, to the best of our knowledge, this is the first report demonstrating significant lipid production by a wild-type strain of *K. marxianus* utilizing a thermally deproteinized whey-based medium without prior lactose enzymatic hydrolysis or genetic modification. From a food and nutraceutical perspective, the use of a non-engineered, GRAS, and QPS yeast strain substantially reduces regulatory barriers, enhances consumer acceptance, and improves the industrial feasibility of food-grade oil production.

The colony morphological and microscopic cellular characteristics of the *K. marxianus* CM3b strain grown on whey agar medium for 48 h at 22 ± 2 °C are shown in Figure 3.

The single colonies displayed typical yeast morphology, appearing circular, white to cream-colored, smooth, creamy, convex, soft (butyrous), and opaque and with entire margins (Figure 3A). Under a light microscope and after Gram staining, the cells were unicellular, ellipsoidal, and Gram-positive, consistent with common yeast characteristics (Figure 3B). ORO staining revealed ellipsoidal cells containing distinct, refractive lipid bodies under the optical microscope (Figure 3C). Furthermore, fluorescence microscopy with a rhodamine filter demonstrated the presence of intracellular neutral lipids, which appeared as red-stained inclusions following ORO staining (Figure 3D).

To further visualize intracellular lipid accumulation in *K. marxianus* CM3b, ORO-stained cells were examined by confocal laser scanning microscopy (Figure 4). The optical image (Figure 4B) shows the cellular morphology, whereas the confocal image (Figure 4A) reveals ORO-associated fluorescence within the cells. The merged image (Figure 4C) demonstrates the spatial correspondence between the cells and the fluorescent signal, providing additional visual evidence of intracellular lipid accumulation in *K. marxianus* CM3b.

### 3.3. Kinetic Study of Cell Growth, Lactose Consumption, and Lipid Production by K. marxianus CM3b

The kinetics of cell growth, lactose consumption, and lipid production during batch cultivation of *K. marxianus* CM3b in whey medium are shown in Figure 5. The growth curve followed the classic pattern of microbial batch culture (Figure 5A). Notably, the absence of a discernible lag phase indicated rapid metabolic adaptation to whey substrate. The exponential growth phase persisted for approximately 16 h of incubation, after which the yeast culture transitioned into the stationary growth phase. No cell death phase was observed during the experimental period.

The maximum biomass concentration (X_max_) of *K. marxianus* CM3b reached 5.50 ± 0.06 g/L at an incubation period of 24 h (Table 4). This biomass concentration was higher than those reported for *K. marxianus* EXF-5288 (4.24 ± 0.04 g/L at 48 h) [65] and *K. marxianus* ATCC 64884 (3.5 g/L at 96 h) [64]. However, it is comparable to that obtained using *K. marxianus* ETP87 (5.48 ± 0.4 g/L) [66], *K. marxianus* CBS 6556, and its recombinant strain KMDB-1 (5.5 g/L) cultivated in whey-based medium for 36 h [67]. These findings demonstrate that *K. marxianus* CM3b achieved substantial biomass production under non-optimized conditions at 22 ± 2 °C, suggesting that further optimization of culture parameters—such as temperature and aeration—could potentially enhance biomass productivity.

The maximum specific growth rate (μ_max_) of *K. marxianus* CM3b in whey was 0.11 h^−1^ (Table 4). The reported μ_max_ values for *K. marxianus* cultivated in whey vary significantly depending on operational parameters, including nutrient availability, pH, temperature, and oxygenation. Under non-optimized conditions, *K. marxianus* typically demonstrates μ_max_ values below 0.3 h^−1^, particularly when the whey is not supplemented with nutrients and incubation temperatures are low or moderate (<30 °C). However, *K. marxianus* is recognized as a fast-growing eukaryote, capable of achieving high μ_max_ values when cultivated in optimized media (0.89–0.99 h^−1^), particularly when supplemented with nutrients—primarily nitrogen—under conditions of high oxygenation and controlled temperatures of approximately 37–40 °C [50,58,61,65,68,69].

The biomass growth and lactose consumption profiles were closely aligned (Figure 5A). The biomass yield of *K. marxianus* CM3b based on lactose consumed (Y_XS_) was 0.38 ± 0.03 g of biomass/g of lactose (Table 4), which was higher than the values reported for several other *K. marxianus* strains (0.07–0.19 g of biomass/g of lactose) [50,65,70] and comparable to that of *K. marxianus* FII 510700 (FRR 1586) (0.37–0.38 g of biomass/g of lactose) [71]. The volumetric biomass productivity of *K. marxianus* reached 5.31 ± 0.11 g biomass/L·d (Table 4), which was higher than the productivity reported for batch cultures of other *K. marxianus* strains isolated and cultured in whey (3.84 g of biomass/L·d) [50].

Lipid accumulation peaked at 24 h (Figure 5B), coinciding with the transition from exponential to stationary growth (Figure 5A). This temporal relationship may reflect changes in nutrient availability and cellular metabolism. In oleaginous yeasts, nitrogen limitation is widely recognized as an important factor promoting lipid accumulation [42,72]. The rapid lipid accumulation observed in *K. marxianus* CM3b may also be associated with lactose assimilation and the predominantly respiratory metabolism generally described for this species under aerobic conditions [54,58]. However, this study did not evaluate nitrogen consumption, ethanol production, the Crabtree phenotype of *K. marxianus* CM3b, or intracellular metabolic fluxes. Therefore, the mechanisms responsible for the early lipid accumulation and its subsequent decline remain to be elucidated.

The maximum lipid concentration (P_max_) achieved by *K. marxianus* CM3b was 2.68 g/L (Table 4), higher than that reported for several yeast species belonging to the genera *Candida*, *Lipomyces*, *Pichia*, *Rhodotorula*, *Trichosporon*, *Yarrowia*, *Debaryomyces*, and *Papiliotrema* (0.27–1.2 g/L). This concentration is comparable to that observed in two strains of *Candida curvatus* (approximately 2.8–3 g/L), although lower than the values reported for *Candida curvatus* NRRL Y-1511 (6.83 ± 0.14 g/L) and *Candida laurentii* UCD 68-201 (5.06 ± 0.28 g/L) cultivated in Ricotta or Mizithra cheese whey [1,73]. Additionally, the lipid concentration obtained with *K. marxianus* CM3b was higher than that of *K. marxianus* IG 1 grown on deproteinized potato-processing wastewater supplemented with 50 g/L lactose (1.74 ± 0.09 g/L) or glycerol (2.16 ± 0.22 g/L) [63].

The lipid yield (Y_px_) of *K. marxianus* CM3b, calculated as grams of lipid per gram of biomass, was 0.49 g/g (49% *w*/*w*) (Table 4). Since microorganisms accumulating more than 20% lipids are classified as oleaginous [26,43,74], *K. marxianus* CM3b clearly exceeds this threshold, confirming its classification as an oleaginous strain under the evaluated conditions. This lipid yield was higher than that reported for *K. marxianus* ATCC 64884 (approximately 0.22 g/g) grown on electro-activated whey [64], as well as the yields observed in several yeast strains from the genera *Candida*, *Debaryomyces*, *Papiliotrema*, *Pichia*, *Rhodotorula*, *Trichosporon*, and *Yarrowia* (0.06–0.33 g of lipids/g of biomass). It is also comparable to the yields achieved by three yeast strains from the genera *Candida*, *Pichia*, and *Rhodotorula* (0.48–0.49 g of lipids/g of biomass) grown on Mizithra or Ricotta cheese whey [1,73]. Although strains such as *C. curvatus* NRRL Y-1511, *C. laurentii* UCD 68-201, *L. starkeyi* NRRL 11557, *R. minuta* UCD 68-280, and *R. toruloides* NRRL Y-17902 demonstrated even higher lipid yields than *K. marxianus* CM3b (0.56–0.79 g of lipids/g of biomass) when grown on Ricotta cheese whey, they required 72 to 96 h of incubation to reach their maximum lipid yield [73]. The shorter cultivation time observed for *K. marxianus* CM3b may be of interest for improving lipid productivity. However, direct comparisons under standardized cultivation conditions and further process optimization are required to establish its relative performance and economic implications.

Although maximum lipid concentration and yield are critical parameters for assessing microorganisms’ suitability for lipid production, these parameters alone are insufficient to select strains with food application potential. Therefore, other parameters such as volumetric lipid productivity, cultivation time, and strain-specific safety frameworks must be considered.

The volumetric lipid productivity of *K. marxianus* CM3b was 2.68 g lipids/L·d (Table 4), which was higher than the values estimated for several yeast strains from the genera *Mortierella*, *Candida*, *Debaryomyces*, *Papiliotrema*, *Cryptococcus*, *Lipomyces*, *Pichia*, *Rhodotorula*, *Trichosporon*, and *Yarrowia* (0.09–2.27 g of lipids/L·d) cultivated in cheese whey with or without supplementation of carbon, nitrogen, chemical growth factors, and/or mineral salt sources [1,73,75,76]. It was also higher than the value reported for *K. marxianus* ATCC 64884 (0.78 g of lipids/L·d) grown on electro-activated whey [64]. However, its productivity was lower than that achieved in systems employing whey pretreatment, such as those for *M. isabellina* DSM 1414 (4.60 g/L·d) [77] and *C. curvatus* KCTC 27583 (4.68 g/L·d) [78], where whey was pretreated with lactase for lactose hydrolysis or subjected to hydrodynamic cavitation under alkaline conditions to enhance the hydrolysis of whey nutrients, thereby increasing their bioavailability and assimilation by yeasts. By contrast, the present study did not use enzymatic lactose hydrolysis, hydrodynamic cavitation, or nutrient fortification beyond standard supplementation, which may reduce operating costs and improve the economic viability of the lipid production process [29].

The specific lipid productivity (R_PX_) of *K. marxianus* CM3b reached 0.506 g of lipids/g of biomass·d (Table 4). This value was higher than those estimated for two strains of *C. curvatus*, two strains of *P. laurentii*, and one strain of *D. hansenii* (0.022–0.065 g of lipids/g of biomass·d) cultivated in Mizithra cheese whey [1], as well as for *K. marxianus* ATCC 64884 (0.463 g of lipids/g of biomass·d) grown on electro-activated whey [64].

### 3.4. Fatty Acid Profile of K. marxianus CM3b

The FA composition of microbial oils is a key determinant of their nutritional value, physicochemical properties, oxidative stability, and overall suitability for food and nutraceutical applications [27,79,80].

The lipid profile of oleaginous microorganisms demonstrates considerable plasticity, shaped by intricate interactions between genetic background and environmental conditions, such as carbon source type and concentration, carbon/nitrogen (C/N) ratio, pH, temperature, oxygenation, and cultivation time [26,72,80,81,82].

In this study, the FA composition of *K. marxianus* CM3b was analyzed after 24 h of cultivation in whey medium, corresponding to the peak of lipid accumulation (Figure 5B). The oil produced by *K. marxianus* CM3b was predominantly composed of FAs with 16 and 18 carbon atoms, whereas those with 14 carbon atoms were present in lower amounts. This fatty acid distribution is consistent with profiles reported for other oleaginous yeast species [83] and closely mirrors the FA composition typical of many edible vegetable oils [34,84], making this microbial oil of potential interest for food-related applications. However, additional physicochemical characterization is required to determine the technological suitability of *K. marxianus* CM3b oil for incorporation into food matrices.

The major FA identified in *K. marxianus* CM3b oil was oleic acid (C18:1), accounting for 53.51% of the total FAs, followed by palmitic acid (C16:0) at 27.72%, palmitoleic acid (C16:1) at 16.05%, stearic acid (C18:0) at 1.53%, and myristic acid (C14:0) at 1.17%. The high oleic acid proportion observed in *K. marxianus* CM3b oil may reflect strain- and cultivation-dependent regulation of fatty acid synthesis and desaturation. Because the present study did not evaluate the activities of enzymes involved in fatty acid biosynthesis, gene expression, or intracellular metabolic fluxes, the current data cannot establish the specific metabolic basis of this fatty acid profile.

Overall, MUFAs made up 69.56% of the total FAs in the sample, whereas saturated fatty acids (SFAs) accounted for 30.42%. Polyunsaturated fatty acids (PUFAs) were not detected under the experimental conditions evaluated. However, this finding should not be interpreted as an intrinsic inability of *K. marxianus* to synthesize PUFAs, since linoleic acid has been reported in oil produced by *K. marxianus* CC1 [85]. Fatty acid composition in yeasts may vary with strain and cultivation conditions [72,80,81,85]. Therefore, although *K. marxianus* CM3b oil showed a high MUFA content, the present results do not establish it as a source of essential PUFAs.

The predominance of oleic acid in *K. marxianus* CM3b oil is of potential nutritional interest because this MUFA is a major component of widely consumed edible oils, including olive and avocado oils [13,18,19,20,86]. Nevertheless, the nutritional suitability of *K. marxianus* CM3b oil requires further evaluation.

The high MUFA proportion in *K. marxianus* CM3b oil may also be of technological interest because fatty acid composition influences the oxidative and thermal behavior of edible oils [87]. However, because the present study did not experimentally determine the oxidative stability and thermal properties of *K. marxianus* CM3b oil, future investigations should evaluate these attributes.

Compared with other oleaginous yeasts cultivated in whey-based media, *K. marxianus* CM3b showed an oleic acid percentage similar to that of *C. curvatus* NRRL Y-1511 cultivated on Ricotta cheese whey (52.8%) [73] but higher than those of several previously reported yeast strains. For example, *C. laurentii* UCD 68-201 cultured on Ricotta cheese whey for 96 h produced 47.16 ± 0.97% oleic acid [73]. Similarly, *C. laurentii* 11 cultivated on whey, whey supplemented with 50 g/L molasses or whey supplemented with 50 g/L molasses, mineral salts, and 0.5 g/L yeast extract yielded 36%, 40.6%, and 32.7% oleic acid at 240 h, 360 h, and 360 h of incubation, respectively [75]. Similarly, *M. isabellina* cultured on Mizithra cheese whey supplemented with 25 g/L lactose for 410 h [76] and *M. isabellina* DSM 1414 cultured in lactase-treated whey for 240 h achieved oleic acid yields of 45.9% and 43.11–45.05%, respectively [77]. Likewise, *C. curvatus* KCTC 27583 cultivated on whey pretreated by hydrodynamic cavitation under alkaline conditions (pH = 12) for 36 h [78] and *K. marxianus* IG 1 grown in YPD medium (20 g/L glucose, 20 g/L peptone, and 10 g/L yeast extract) [63] achieved oleic acid yields of 45.3% and 34.87%, respectively.

Notably, these studies typically required whey pretreatment, lactose hydrolysis, supplementation with additional carbon sources, growth factors, and/or salts, as well as extended fermentation periods ranging from 72 to 410 h. By contrast, *K. marxianus* CM3b produced 53.51% oleic acid in just 24 h of cultivation using thermally deproteinized whey medium without prior enzymatic lactose hydrolysis. While these comparisons provide useful context across studies, differences in substrate composition, pretreatment methods, nutrient supplementation, cultivation conditions, and fermentation times indicate that direct comparisons should be interpreted with caution.

### 3.5. Fatty Acid Productivities

In addition to FA composition, productivity is a crucial parameter for evaluating process performance and industrial relevance. Both volumetric (R_FA_) and specific (R_FAX_) productivities of individual FAs were determined at the point of maximum lipid accumulation (24 h), as shown in Figure 6.

Among the fatty acids analyzed, oleic acid demonstrated the highest volumetric and specific productivity, reaching 59.75 ± 0.9 mg/L·h and 11.27 ± 0.32 mg/g of biomass·h, respectively. Palmitic acid followed with 30.96 ± 0.56 mg/L·h and 5.84 ± 0.16 mg/g of biomass·h, whereas palmitoleic acid achieved 17.93 ± 0.33 mg/L·h and 3.39 ± 0.09 mg/g of biomass·h. By contrast, stearic acid (1.71 ± 0.03 mg/L·h and 0.32 ± 0.009 mg/g of biomass·h) and myristic acid (1.31 ± 0.02 mg/L·h and 0.247 ± 0.007 mg/g of biomass·h) were produced at substantially lower rates.

Despite the critical role of productivity in evaluating process efficiency, optimizing operational parameters and fermentation time, and facilitating scale-up, literature reports on FA productivity in whey-based systems remain scarce. Therefore, oleic acid productivity was estimated from published data to allow comparison. The volumetric and specific oleic acid productivities achieved by *K. marxianus* CM3b were higher than those reported for *C. curvatus* NRRL Y-1511 and *C. laurentii* UCD 68-201 grown on Ricotta cheese whey, which showed volumetric productivities of 50.306 and 37.82 mg/L·h and specific productivities of 4.67 and 3.38 mg/g of biomass·h, respectively [73]. Similarly, these parameters were higher than the values documented for *M. isabellina* ATHUM 2935 cultured on Mizithra cheese whey, which reached 3.92 mg/L·h and 0.093 mg/g of biomass·h [76]. Furthermore, the *K. marxianus* CM3b values were higher than those obtained with *C. laurentii* 11 under different whey-based conditions, including crude whey (1.905 mg/L·h and 0.417 mg/g of biomass·h), whey supplemented with 50 g/L of sugar cane molasses (1.70 mg/L·h and 0.103 mg/g of biomass·h), and whey supplemented with molasses, yeast extract, and mineral salts (2.69 mg/L·h and 0.007 mg/g of biomass·h) [75].

The oleic acid productivity achieved by *K. marxianus* CM3b after 24 h of cultivation, together with its ability to utilize whey lactose without prior enzymatic hydrolysis, supports further investigation of this wild-type strain as a potential microbial source of oleic acid-rich oil for food-related applications.

Comparisons of *K. marxianus* CM3b with previously reported oleaginous microorganisms should be interpreted cautiously because the studies differ in strain, substrate composition, cultivation conditions, culture scale, and fermentation time. Therefore, these literature comparisons provide context rather than evidence of direct superiority, and head-to-head comparison under identical cultivation and experimental conditions would be required to establish relative strain performance.

### 3.6. Comparison of the Fatty Acid Profile of K. marxianus CM3b Oil with Those of Vegetable Oils

The FA profiles of oleaginous yeast closely resemble those of vegetable oils, being rich in SFAs and MUFAs with 16- and 18-carbon chains [81,88,89]. The FA composition of oils is crucial in determining their physical properties, functional characteristics, and various industrial applications [81].

To contextualize the potential food applications of *K. marxianus* oil, its FA profile was compared with those of several widely used vegetable oils in the food industry, including olive (*Olea europaea*), avocado (*Persea americana* Mill), soybean (*Glycine max*), peanut (*Arachis hypogaea*), sunflower (*Helianthus annuus*), and palm (*Elaeis guineensis*) oils. The FA compositions of these vegetable oils were sourced from the established literature [34,84,86].

Radial plot analysis demonstrated that the FA profile of *K. marxianus* CM3b oil closely aligns with those of avocado (Figure 7A) and olive (Figure 7B) oils, both characterized by high oleic acid content and moderate palmitic acid levels. By contrast, soybean, peanut, and sunflower oils are characterized by elevated concentrations of linoleic acid (C18:2) (Figure 7C,D,F), a PUFA that was not detected in *K. marxianus* CM3b oil. Palm oil, although rich in SFAs, differed from *K. marxianus* CM3b oil owing to its greater proportion of palmitic acid and lower levels of MUFAs (Figure 7E).

The compositional similarity of FAs between *K. marxianus* CM3b oil and avocado and olive oils is particularly significant, considering their well-established health benefits and premium market value. This similarity suggests potential interest in developing microbial oils for food-related applications; nevertheless, further studies are needed to demonstrate equivalent nutritional, physicochemical, or functional properties.

Furthermore, microbial oil production offers distinct advantages over traditional plant-based sources, as it is independent of agricultural land use, seasonal fluctuations, and climatic limitations. This independence enables a controlled and potentially more sustainable supply of high-oleic acid oils [27,28,29]. In addition, valorizing cheese whey as a substrate for microbial oil production closely aligns with circular bioeconomy principles. However, appropriate sustainability assessments are needed to establish the environmental benefits of the proposed process.

Although the high proportion of MUFAs, particularly oleic acid, in *K. marxianus* CM3b oil and the similarity of its fatty acid profile to those of conventional edible oils such as olive and avocado oils are compositional characteristics of potential interest for food applications, fatty acid composition alone is insufficient to establish the suitability of *K. marxianus* CM3b oil as a food ingredient. Additional physicochemical characterization, including acid value, peroxide value, oxidative stability, and thermal properties, as well as sensory evaluation and strain-specific safety assessment, will be required to establish its suitability for food applications.

Because the present study used reconstituted, thermally deproteinized whey and did not include untreated raw industrial whey as a control, the observed performance cannot be directly extrapolated to untreated industrial whey streams. Therefore, future studies should evaluate *K. marxianus* CM3b using actual industrial whey streams with different compositions and degrees of processing.

Furthermore, because the present results were obtained from non-optimized batch cultures in shake flasks with a 200 mL working volume, they cannot be directly extrapolated to controlled bioreactor conditions or industrial-scale operation. Future process-development studies should address oxygen transfer and dissolved oxygen requirements, mixing and hydrodynamic conditions, pH and temperature control, foam formation and control, optimization of cultivation and feeding strategies, lipid recovery and extraction, and upstream and downstream processing costs.

## 4. Conclusions

A newly isolated wild-type yeast strain, identified as *Kluyveromyces marxianus* CM3b, produced oleic acid-rich microbial oil from a thermally deproteinized whey-based medium under non-optimized batch cultivation conditions. Maximum lipid accumulation was observed after 24 h, with a lipid yield of 49% (*w*/*w*) and a volumetric lipid productivity of 2.68 g/L·d. The resulting oil contained 53.51% oleic acid and exhibited compositional similarities to olive and avocado oils. *K. marxianus* CM3b’s ability to utilize whey lactose without prior enzymatic hydrolysis, along with its rapid lipid accumulation and high oleic acid content, supports its potential for further development as a microbial oil-producing strain. Considering the established use of *K. marxianus* in food fermentations, these findings are particularly relevant to investigating microbial oils for potential food applications. However, the safety of *K. marxianus* CM3b and the suitability of its oil for human consumption remain to be established.

## Figures and Tables

**Figure 1:**
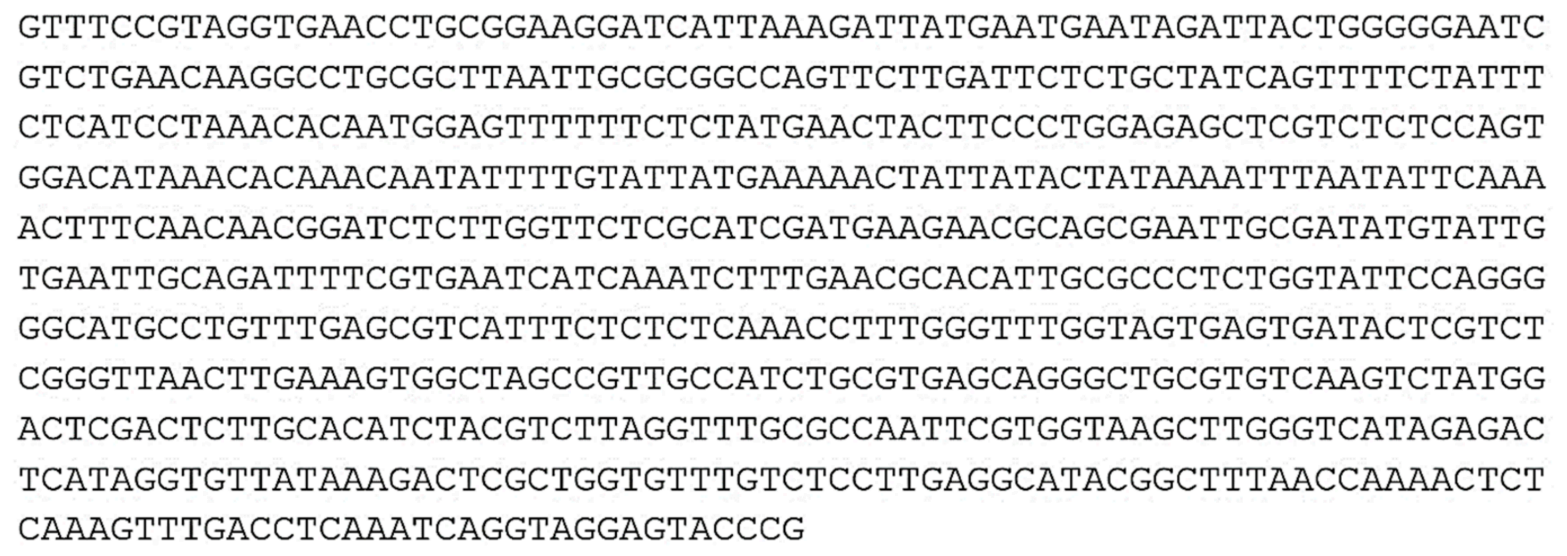
Sequence of the 16S-ITS1-5.8S-ITS2-26S region of the rDNA of the yeast isolate CM3b.

**Figure 2:**
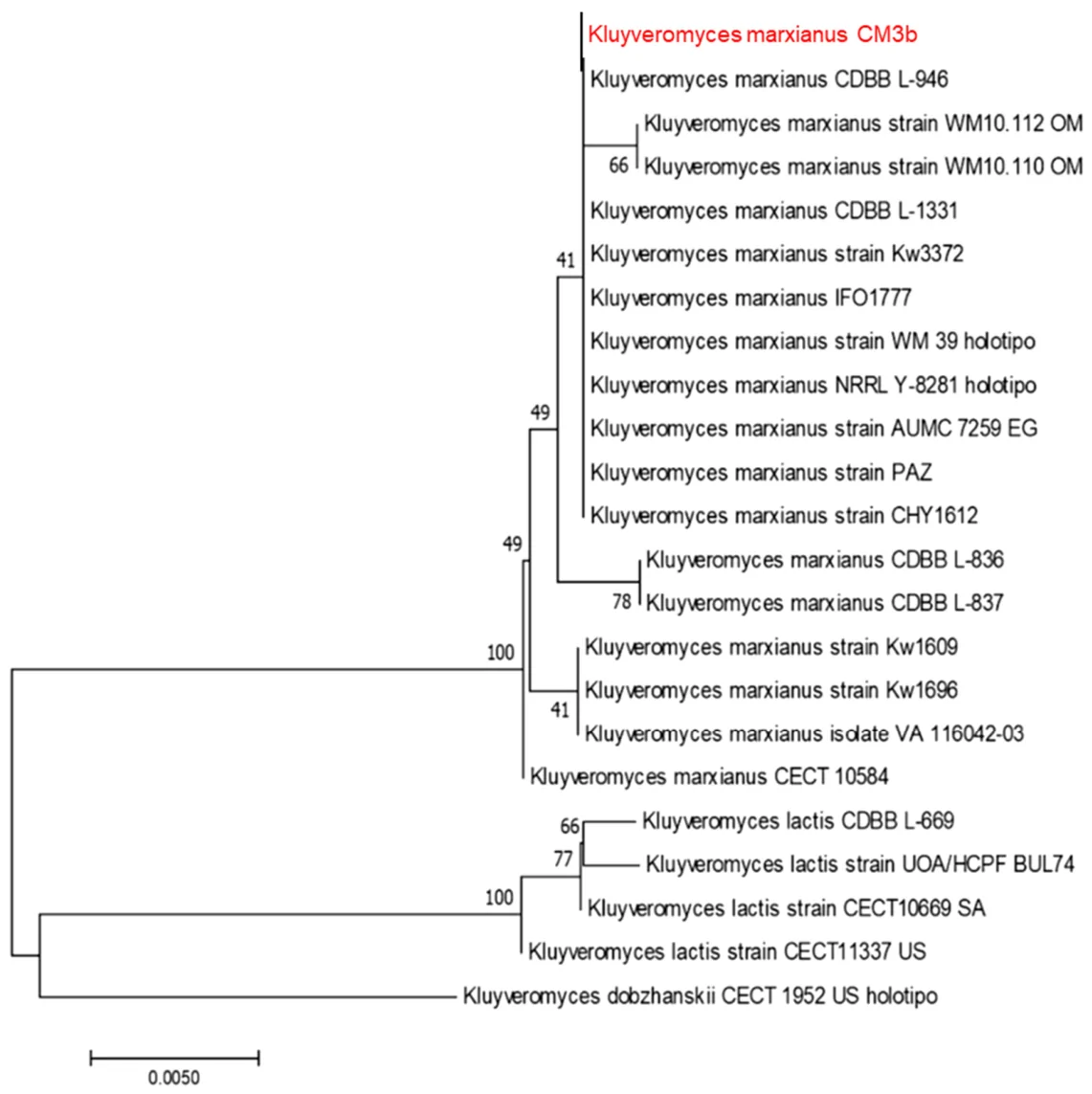
Dendrogram demonstrating the phylogenetic position of isolate CM3b (shown in red) among *K. marxianus* yeast strains.

**Figure 3:**
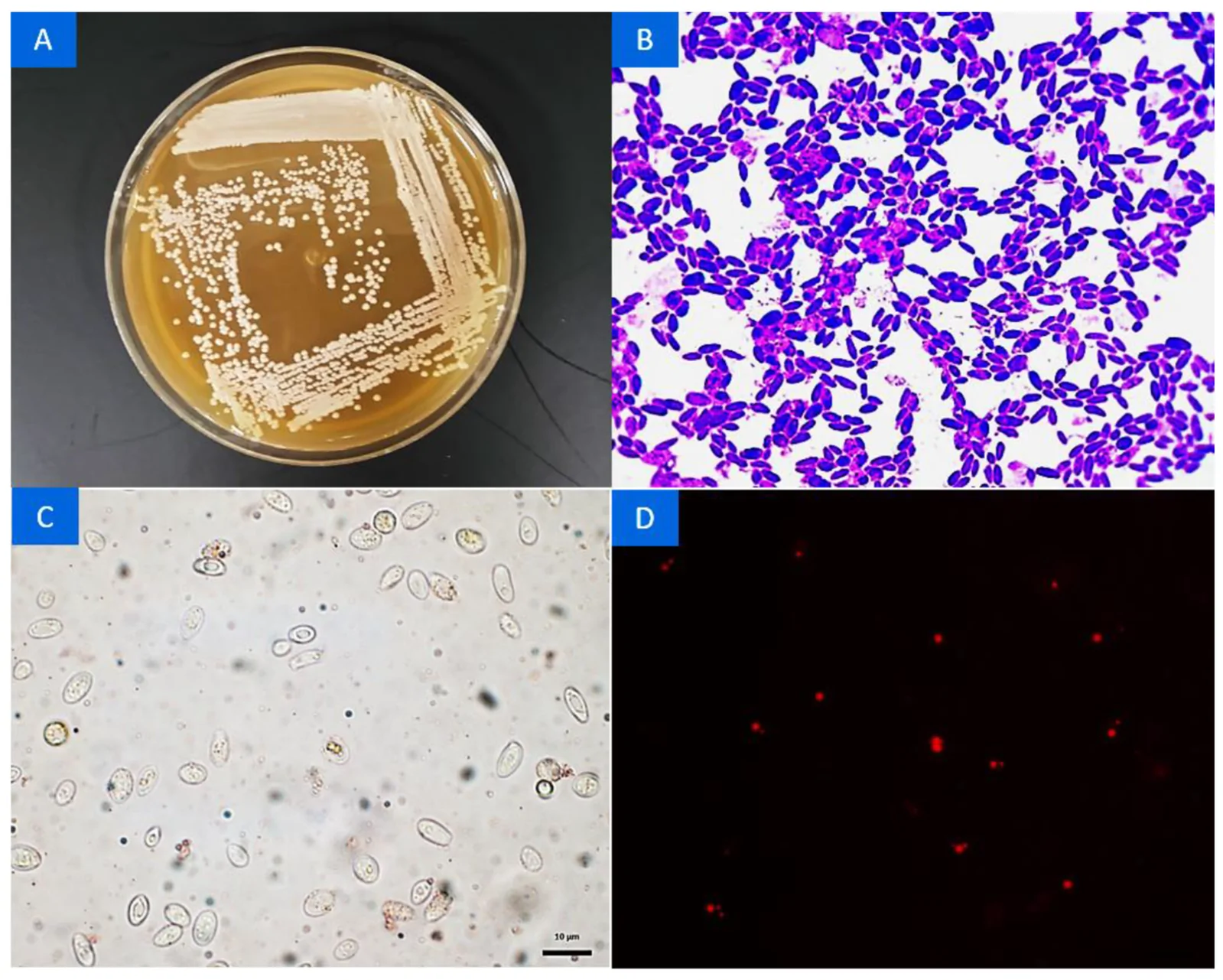
Characteristics of the colonial and microscopic morphology of *K. marxianus* CM3b grown on whey agar medium for 48 h at 22 ± 2 °C. (**A**) Colonial morphology; (**B**) microscopic morphology under an optical microscope after Gram staining; (**C**) microscopic morphology under an optical microscope after ORO staining; (**D**) fluorescence micrograph using a rhodamine filter of intracellular neutral lipid after staining with ORO.

**Figure 4:**
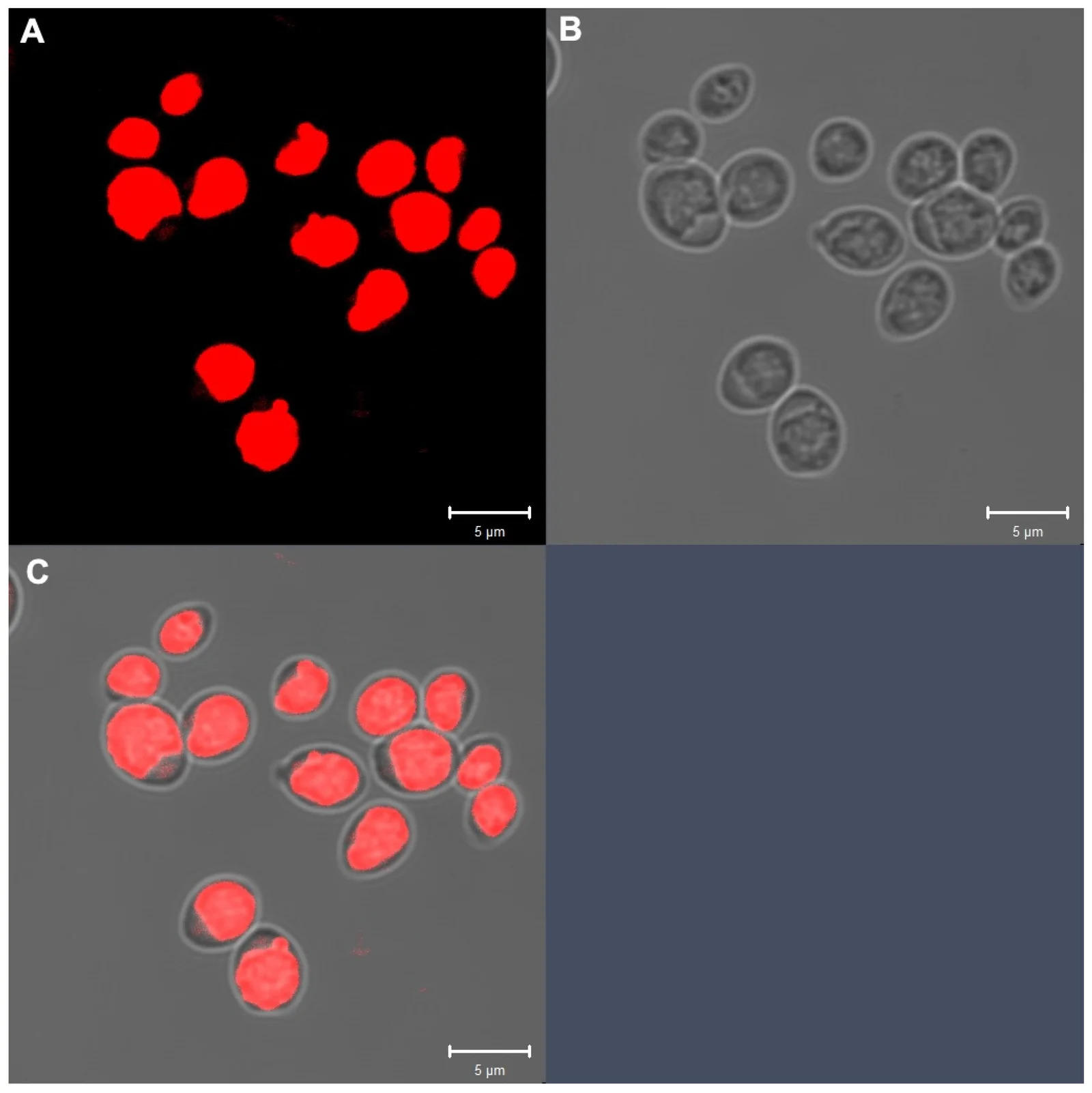
Confocal laser scanning microscopy of Oil Red O-stained *Kluyveromyces marxianus* CM3b grown in whey medium. (**A**) Confocal fluorescence image, (**B**) optical microscopy image, and (**C**) merged image showing intracellular lipid accumulation. Images were acquired at 900× magnification.

**Figure 5:**
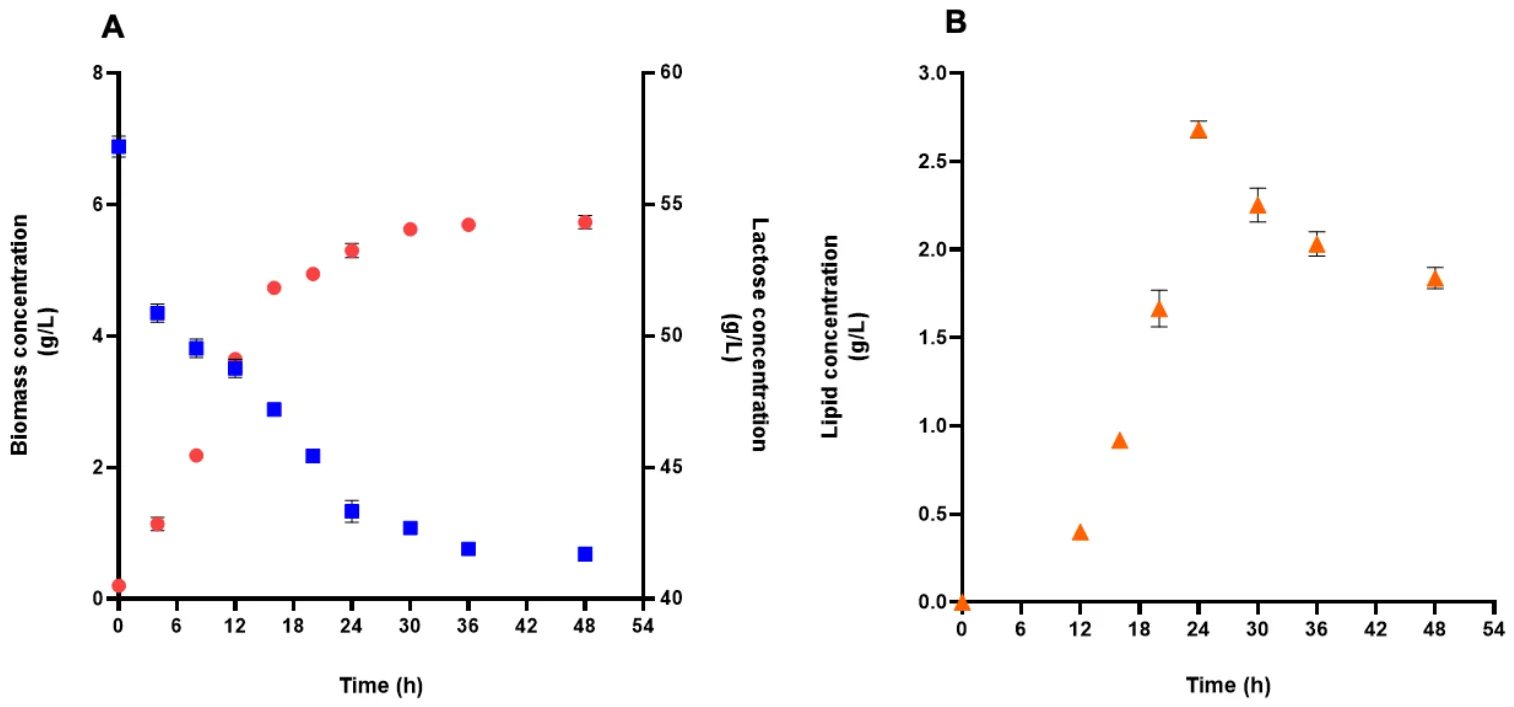
Kinetics of (**A**) (●) cell growth, (■) lactose consumption, and (**B**) (▲) lipid production by *K. marxianus* CM3b.

**Figure 6:**
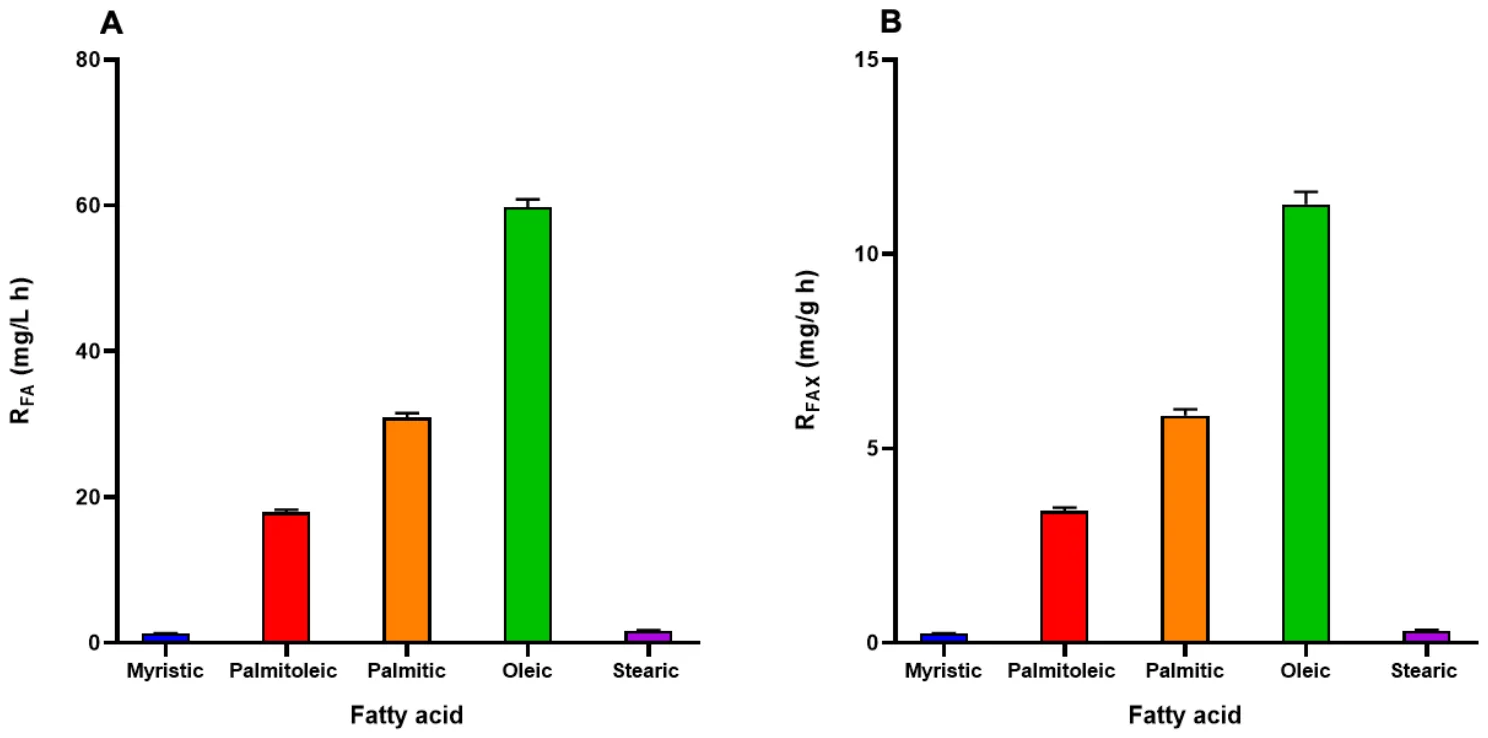
(**A**) Volumetric (R_FA_) and (**B**) specific (R_FAX_) productivities of fatty acids of the *K. marxianus* CM3b oil cultured on cheese whey.

**Figure 7:**
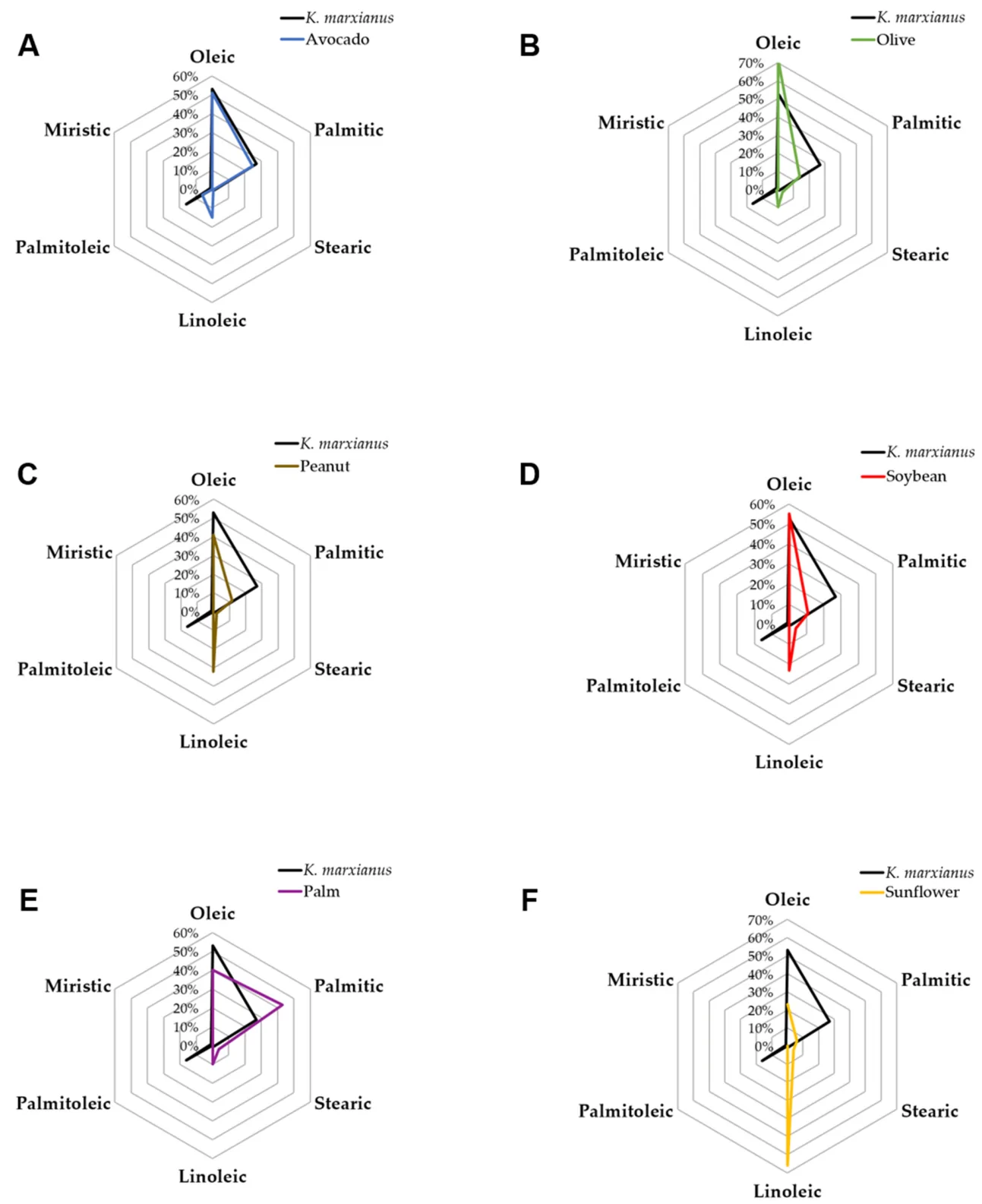
Comparison between the fatty acid composition of *K. marxianus* CM3b oil cultivated on cheese whey for 24 h and different vegetable oils extracted from (**A**) avocado, (**B**) olive, (**C**) peanut, (**D**) soybean, (**E**) palm, and (**F**) sunflower.

**Table 1:** Isolation sources of microorganisms.

Identification Code	Isolation Source	Geographical Coordinates
CM1	Spoiled unpasteurized whole milk	19°47′23.1″ N 99°03′42.4″ O
CM2	Spoiled pasteurized cream	N.A.
CM3	Spoiled unpasteurized whey	19°30′04.6″ N 99°08′40.8″ O
CM4	Spoiled pasteurized whole milk	N.A.
CM5	Corn farm soil	19°45′34.0″ N 99°08′18.2″ O
CM6	Alfalfa farm soil	19°47′27.1″ N 99°10′02.8″ O

N.A. = not applicable.

**Table 2:** MALDI-TOF MS proteomic identification of microbial isolates.

Microbial Isolate	Family Name	Microbial Species	% of Similarity
CM1a	Saccharomycetaceae	*Candida krusei*	99.99
CM2a	Saccharomycetaceae	*Candida krusei*	99.99
CM3a	Saccharomycetaceae	*Candida tropicalis*	99.99
CM3b	Saccharomycetaceae	*Candida kefyr*	99.99
CM5a	Endomycetaceae	*Pichia fabianii*	99.99

**Table 3:** Total lipid content of microbial isolates.

Microbial Isolate		Lipid Content (%)		
		24 h	48 h	72 h
*Candida krusei*	CM1a	25.31 ± 1.09	33.14 ± 2.31	11.98 ± 2.69
*Candida krusei*	CM2a	24.15 ± 1.97	32.67 ± 3.31	12.83 ± 1.67
*Candida tropicalis*	CM3a	32.40 ± 2.28	41.77 ± 3.12	27.02 ± 2.18
*Candida kefyr*	CM3b	50.03 ± 2.96	33.16 ± 4.19	21.50 ± 2.54
*Pichia fabianii*	CM5a	13.25 ± 0.93	16.25 ± 0.91	8.33 ± 0.42

**Table 4:** Kinetic parameters of cell growth and lipid production by *K. marxianus* CM3b grown on a whey substrate.

Parameter	Value
Maximum biomass concentration, X_max_ (g/L)	5.50 ± 0.06
Maximum specific growth rate, μ_max_ (h^−1^)	0.11 ± 0.001
Biomass yield based on lactose consumed, Y_xs_ (g of biomass/g of lactose)	0.38 ± 0.03
Volumetric biomass productivity, R_X_ (g of biomass/L·d)	5.31 ± 0.11
Maximum lipid concentration, P_max_ (g/L)	2.68 ± 0.08
Lipid yield based on biomass produced, Y_px_ (g of lipid/g of biomass)	0.49 ± 0.02
Volumetric lipid productivity, R_P_ (g of lipid/L·d)	2.68 ± 0.08
Specific lipid productivity, R_PX_ (g of lipids/g of biomass·d)	0.506 ± 0.01

## Data Availability

The original contributions presented in this study are included in the article/Supplementary Material. Further inquiries can be directed to the corresponding author.

## Supplementary Materials

The following supporting information can be downloaded at: https://www.mdpi.com/article/10.3390/foods15193556/s1, Figure S1: Characteristics of the colonial and microscopic morphology of *K. marxianus* CM3b grown on whey agar medium for 48 h at 22 ± 2 °C. (A) Colonial morphology; (B) Microscopic morphology under an optical microscope after Gram staining; (C) Microscopic morphology under an optical microscope after ORO staining; (D) Fluorescence micrograph using a rhodamine filter of intracellular neutral lipid after staining with ORO; Table S1: Microbial isolates capable of growing and accumulating oils from whey as a source of carbon and energy.

## Abbreviations

The following abbreviations are used in this manuscript:

MUFA	Monounsaturated fatty acid
FA	Fatty acid
FAME	Fatty acid methyl ester
ORO	Oil Red O
GRAS	Generally Recognized as Safe
QPS	Qualified Presumption of Safety
PUFA	Polyunsaturated fatty acid
SFA	Saturated fatty acid
