# Supplementary material for: Newly Isolated Kluyveromyces marxianus Strain Capable of Producing Oleic Acid-Rich Oil for Human Consumption from Whey
Source: Foods. 2026 Oct 7;15(19):3556. doi: 10.3390/foods15193556 (PMC13664798; doi:10.3390/foods15193556)
Supplement: Supplementary file 1 [file foods-15-03556-s001.zip › foods-4549453-supplementary.pdf]

Supplementary Material

Newly Isolated *Kluyveromyces marxianus* Strain Capable of Producing Oleic Acid-Rich Oil for Human Consumption from Whey

Table S1. Microbial isolates capable of growing and accumulating oils from whey as a source of carbon and energy.

Identification code of isolation source	Number of microbial isolates capable of growing on whey	Number and name(s) of microbial isolates capable of accumulating lipids from whey
**CM1**	3	1 (CM1a)
**CM2**	3	1 (CM2a)
**CM3**	2	2 (CM3a and CM3b)
**CM4**	2	0
**CM5**	2	1 (CM5a)
**CM6**	1	0

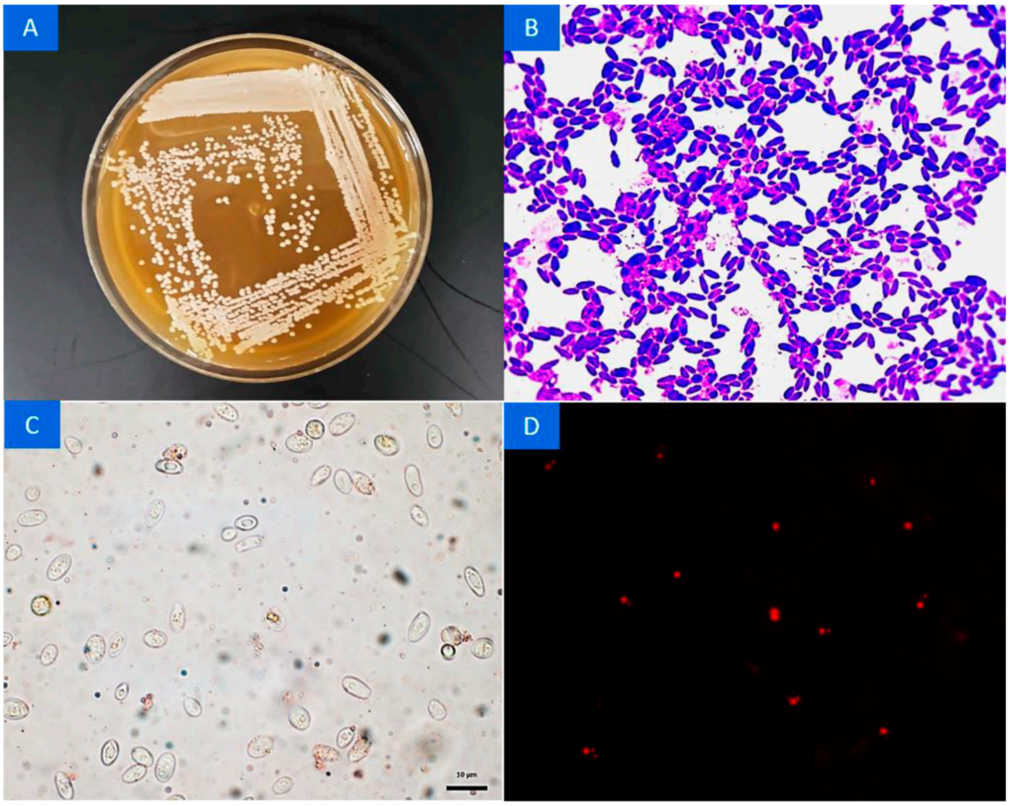

Figure S1. Characteristics of the colonial and microscopic morphology of *K. marxianus* CM3b grown on whey agar medium for 48 h at 22 ± 2°C. (A) Colonial morphology; (B) Microscopic morphology under an optical microscope after Gram staining; (C) Microscopic morphology under an optical microscope after ORO staining; (D) Fluorescence micrograph using a rhodamine filter of intracellular neutral lipid after staining with ORO.

The single colonies displayed typical yeast morphology, appearing circular, white to cream-colored, smooth, creamy, convex, soft (butyrous), opaque, and with entire margins (Figure S1A). Under a light microscope and after Gram staining, the cells were unicellular, ellipsoidal, and Gram-positive, consistent with common yeast characteristics (Figure S1B). ORO staining revealed ellipsoidal cells containing distinct, refractive lipid bodies under the optical microscope (Figure S1C). Furthermore, fluorescence microscopy with a rhodamine filter demonstrated the presence of intracellular neutral lipids, which appeared as red-stained inclusions following ORO staining (Figure S1D).
